# Gas Turbine Blade Failures Repaired Using Laser Metal Additive Remanufacturing

**DOI:** 10.3390/ma18245590

**Published:** 2025-12-12

**Authors:** Changjun Chen, Min Zhang, Haodong Liu, Qingfeng Yang

**Affiliations:** 1 Laser Processing Research Center, School of Mechanical and Electric Engineering, Soochow University, Suzhou 215131, China; 2 Naval Aviation University Qingdao Branch, Qingdao 266041, China; mdlhd@sina.com (H.L.); fengqingyang882025@163.com (Q.Y.)

**Keywords:** gas turbine blade, erosion, crack, damage, in situ repair, laser cladding, laser metal additive remanufacturing, repair

## Abstract

The production of reliable turbo machinery, particularly gas turbine blades, is a major global challenge. This capability serves as a key indicator of a nation’s industrial base, technological prowess, and comprehensive strength. Critical components in aircraft engines and gas turbines operate under extreme conditions, including high temperatures, high pressures, and substantial mechanical stresses. Consequently, there is a growing urgency to develop cost-effective and time-efficient repair strategies to enhance engine performance and efficiency. However, many mission-critical parts, especially high-pressure (HP) blades, are prone to severe damage. Moreover, taking equipment offline for blade maintenance and repair is a time-consuming process. It is also highly costly to restore these essential components to full functionality. Since 1996, researchers have focused on applying laser metal deposition (LMD) additive manufacturing technology for high-performance repair and remanufacturing of aerospace engines and industrial gas turbine (IGT) blades. Empirical studies have demonstrated that depositing a high-quality, erosion-resistant protective coating on the leading edge of HP blades effectively extends the service life of turbine blades in both aircraft engines and industrial gas turbines. This study systematically outlines the technical workflow of the proposed methodology and provides a concise perspective on emerging development trends.

## 1. Introduction

Throughout the history of aerospace engineering, the selection and use of materials for manufacturing aircraft and helicopters have been driven by the need to meet strict performance requirements. These requirements mainly include high specific strength, good weight efficiency, excellent energy absorption, and long-term structural durability [1]. Aeroengines are among the most critical components in the development of the aviation and aerospace industries. Aeroengines are essential systems for energy and power conversion. Their complex, multi-disciplinary manufacturing and integration of advanced technologies make them a benchmark for a nation’s industrial strength.

Aircraft are often described as the crown of modern industry, while aeroengines and gas turbine engines are considered the brightest jewel in that crown. To improve aeroengine performance, effective measures must be taken to enhance the performance of high-value-added, mission-critical components such as turbine blades.

Gas turbine blades are vital components in modern industry. They are critical to the aerospace and defense sectors—including aircraft, warships, and missiles—as well as to key civilian industries such as power generation, petroleum, and chemicals [1]. The pursuit of a high thrust-to-weight ratio, improved combustion efficiency, and higher speeds has made aeroengines a key research focus in the rapidly evolving aviation industry. Modern gas turbines operate under extreme conditions, such as high temperatures and high pressures, to achieve greater energy output and improved conversion efficiency [1]. As a result, continuous increases in turbine inlet temperature (TIT) and compressor discharge pressure have made the service environment of hot-section turbine blades increasingly severe [2,3,4,5,6,7].

In service, defects on superalloy turbine blades often result from local degradation caused by high creep, fatigue, pressure, oxidation, and erosion. The most straightforward solution is to replace the old components with new ones.

However, the high cost of materials and fabrication for Ni-based superalloy components, together with the expense of replacement, has encouraged the development of repair, remanufacturing, or rejuvenation processes. These processes help restore turbine blades and extend their service life.

This is particularly important for land-based gas turbine engines and single-crystal superalloy blades. A single blade in such applications is capital-intensive and can cost tens of thousands of dollars [8].

The regeneration and repair of aero-engines also require a comprehensive overhaul. Turbine component regeneration has become a critical focus for MRO providers in both the aviation and industrial gas turbine sectors. Empirical data show that propulsion system maintenance accounts for about 8% of the total aircraft operating cost (AOC). Module overhaul represents the largest part of this expenditure. Among these costs, airfoil-related operations make up nearly 50%, primarily due to high-pressure turbine (HPT) blade replacements.

First-stage high-pressure turbine (HPT) blades are among the most thermomechanically stressed components in modern turbofan engines. They endure extreme centrifugal loads exceeding 10,000× *g*, combustion gas temperatures above 1600 °C, and thermal gradients greater than 500 °C/cm. These severe operating conditions lead to frequent refurbishment or replacement needs. For example, a full set of Stage 1 rotor blades for the CF6-80C2 powerplant has an OEM price approaching USD 500,000 (as of 2023) [9,10]. The global market for blade repairs is substantial. Statistics show that servicing jet engines in aircraft and other flying vehicles constitutes at least 30% of the total maintenance cost of an aircraft [11].

Significant costs are associated with precision casting, high-temperature protective coatings, precision machining, and the use of relatively rare elements and refractory metals. When blades are deployed in military aircraft, their failure can lead to substantial economic losses and reduced military strategic value.

Turbine blade defects represent a long-term concern that requires assessment throughout the entire service life of aero-engines. Moreover, preventing blade failure and extending blade service life are critically important issues for aircraft engines and gas turbines. As critical components, the service performance and lifespan of these blades are strongly influenced by the defects they encounter.

With the ongoing global shift toward energy transformation and sustainable development, the remanufacturing of damaged and retired blades has gained increasing worldwide attention. As an enabler of the circular economy, blade remanufacturing represents an effective approach to maximizing a product’s service life and resource utilization.

Early studies have explored the use of traditional methods—such as gas tungsten arc welding (GTAW) [12] and plasma transferred arc welding (PTAW)—for repairing and remanufacturing damaged blades [13]. However, these conventional techniques suffer from several drawbacks, including high heat input into the base material, hot cracking in deposited layers, and poor bonding strength between the coating and substrate. Moreover, modern engines increasingly incorporate blades and components that are difficult or impossible to refurbish using traditional welding methods. Consequently, more advanced repair and remanufacturing processes are required. Such challenging blades or damaged parts often lead to large heat-affected zones (HAZs), high substrate dilution rates, strain-age cracking, and distortion [14,15,16,17,18].

As a revolutionary technology for manufacturing and remanufacturing metal parts, laser powder bed fusion (LPBF) and laser metal deposition (LMD) have been developed and are attracting global interest [19,20,21]. LMD is also referred to as laser cladding or laser additive remanufacturing deposition.

Several researchers have explored the application of laser metal deposition (LMD) technology in remanufacturing. LMD encompasses processes such as laser direct deposition and laser cladding. In a comprehensive review [20], some authors systematically analyzed the machining technologies commonly used in engine blade regeneration. The authors of reference [13] introduced a new technique for compressor blisk regeneration and an innovative turbine blade regeneration process. Both methods were developed within the Collaborative Research Centre (CRC) 871 project [22].

In 2018, a team of researchers introduced a damage detection and reconstruction algorithm for repairing compressor blades using direct metal deposition (DMD) [23]. However, the widespread adoption of such repair technologies remains limited. This is mainly due to the constraints of traditional manual remanufacturing processes, which often fail to ensure consistent part quality. To overcome this challenge, this study applies reverse engineering to identify and extract the repair volume of damaged blades. A corresponding toolpath for additive remanufacturing is then generated. This method enables automated damage detection and geometric reconstruction for jet engine blade repair.

To demonstrate the process, Ti-6Al-4V powder was deposited onto a damaged area using a laser-assisted DMD process. Experimental results verify that the proposed algorithm is suitable and efficient for the automated repair of curved blades. In a related study [24], the authors successfully repaired defective voids in turbine airfoils. They used a novel semi-automated geometric reconstruction algorithm combined with laser direct deposition.

A review of existing literature shows that research on LMD-formed layers for damaged blade repair remains limited, particularly in the context of laser additive manufacturing (LAM) and reverse engineering (RE) applied to high-pressure blades [19,20,21,22,23,24,25,26,27,28,29].

A comprehensive analysis of gas turbine blade failure cases further reveals a significant research gap in repair and remanufacturing methodologies for damaged or failed components. Most published studies focus mainly on failure modes and their causes, while offering few practical solutions. Given the high economic value of these blades, substantial efforts have been made to develop suitable repair or remanufacturing techniques. However, the lack of a validated and reliable repair framework means that successful blade restoration still demands broad expertise and extensive practical experience.

Therefore, further research is essential to establish effective solutions and preventive strategies for blade failure and repair.

Although considerable research has focused on characterizing and analyzing failures in gas turbine blades, comprehensive reviews on repair and remanufacturing methods for laser-clad turbine blades are still lacking. This work investigates the application of laser additive remanufacturing—specifically laser cladding—for restoring nickel-based turbine blades. The study makes three main contributions:

(1) a systematic analysis of key factors leading to blade failure;
(2) a thorough evaluation of research methods used in laser additive remanufacturing;
(3) a detailed discussion of process optimization techniques for repaired components.

Despite its considerable potential, the widespread adoption of laser metal deposition (LMD) for blade repair still faces critical challenges.

First, it remains difficult to achieve consistent metallurgical bonding and defect-free deposits—free from cracks and porosity—on complex curved surfaces such as turbine blade tips. This is mainly due to uneven heat dissipation and residual stress accumulation.

Second, the absence of standardized process parameters for different blade materials, including single-crystal superalloys, hinders process repeatability and reproducibility.

Third, repaired blades often underperform new components in critical areas like fatigue life and oxidation resistance. This performance gap raises serious concerns about their long-term reliability.

Moreover, the high cost of in situ monitoring and adaptive control systems presents a barrier to industrial scalability.

Addressing these issues will require advances in multi-physics process modeling, hybrid post-processing methods, and cost-effective real-time quality assurance.

Ultimately, turbine blade remanufacturing supports the transition toward a circular economy and sustainable development by significantly reducing the consumption of virgin materials and overall resource use.

In the present study, a flowchart illustrating the laser metal additive remanufacturing processes used in our laboratory is briefly introduced. Additionally, an improved turbine blade remanufacturing process developed by our team is presented. This work also offers a concise outlook on future developments and emerging trends in laser additive remanufacturing technologies.

## 2. Factors Leading to the Failure of Gas Turbine Blades

Thermo-mechanical fatigue caused by cyclic loading and extreme thermal gradients is the primary failure mechanism in gas turbine blades. This leads to progressive crack growth, surface spallation, and microstructural degradation. Numerous review papers and research studies addressing this issue are available in major academic databases, including ScienceDirect, IEEE Xplore, Springer, and MDPI [30]. A bibliometric analysis was performed using R-Studio on approximately 1500 articles retrieved from Scopus. The search identified 863 relevant publications on “failure analysis of wind turbine blades” and 629 on “failure analysis of gas turbine blades” [31]. Substantial research has been conducted on gas turbine blade failure analysis to develop effective prevention strategies [31]. The main causes of blade damage include surface degradation, wear, material separation, and structural deformation. These issues typically result from thermomechanical stresses and chemical interactions [30,31].

**Figure 1:**
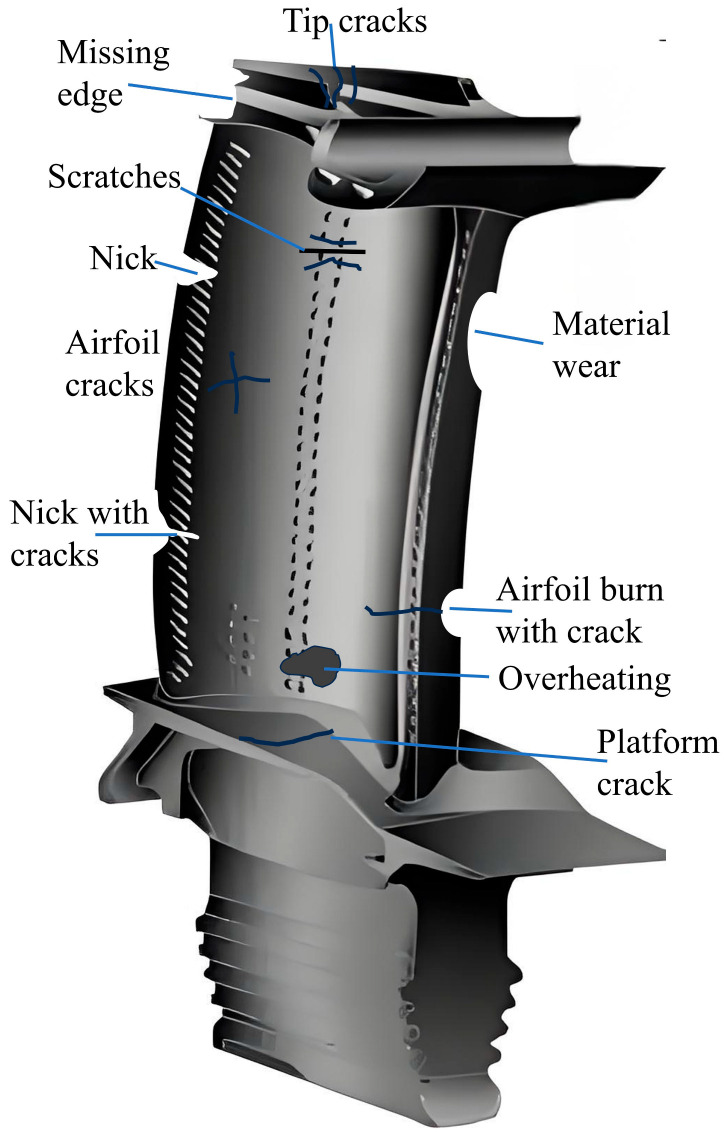
Schematic illustration of cascade failure progression in a gas turbine system initiated by blade integrity compromise.

Figure 1 shows common failure modes found in gas turbine blades. Classifying blade damage is challenging due to inconsistent terminology used by engine manufacturers and maintenance, repair, and overhaul (MRO) providers. For example, failures caused by high temperatures may be described differently in the literature [19].

**(1) Microstructure changes**: Microstructural evolution occurs in response to significant thermal variations, particularly under overheating conditions (Figure 1). This transformation is characterized by phase boundary migration, grain coarsening, and precipitate dissolution, as demonstrated in the micrographs of Figure 1.

**(2) Oxidation:** Oxidation occurs when the blade material undergoes chemical reactions with ambient air or sulfides, typically resulting in coating degradation and material loss. This oxidative process is characterized by the formation of oxide scales (e.g., Cr_2_O_3_ and Al_2_O_3_); the depletion of protective elements in subsurface layers; and the progressive thinning of the base material.

**(3) Cracks**: Cracks will be formed due to high-temperature and high-tensile stresses caused by thermal fatigue. Some types of cracks are shown in Figure 1.

**Abrasion**: The blade surface will be removed due to abrasion by sand or small particles; this is not shown in Figure 1.

**(4) Deformation**: Blade deformation can occur through one of the following two primary mechanisms: (a) foreign object damage (FOD) or (b) creep deformation under sustained thermal–mechanical loading. These deformation modes are characterized by impact-induced damage and creep deformation. The former includes localized plastic strain, surface dent formation, and subsurface microcrack initiation. The latter involves grain boundary sliding, cavitation damage, and progressive airfoil distortion.

**(5) Entire breakage or fracture**: Catastrophic blade fracture is the most severe failure mode in turbine systems. This failure typically originates from one of three mechanisms: foreign object damage (FOD)-induced crack propagation, thermomechanical fatigue crack nucleation and growth, or creep rupture under sustained operational loads. This failure mechanism exhibits the following three characteristics: critical stress intensity exceeding material toughness, transgranular or intergranular fracture surfaces, and potential secondary damage to adjacent components.

## 3. Remanufacturing Process

### 3.1. Remanufacturing Strategy

**Figure 2:**
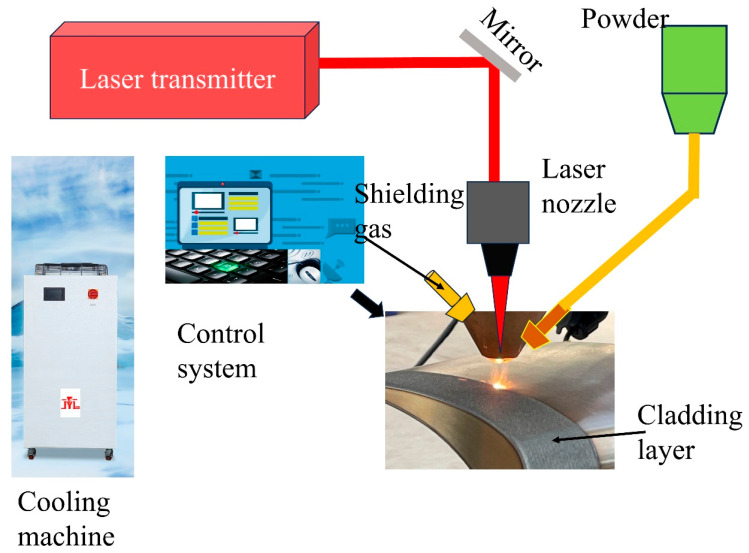
Schematic view of the laser metal deposition (laser cladding) remanufacturing process.

**Figure 3:**
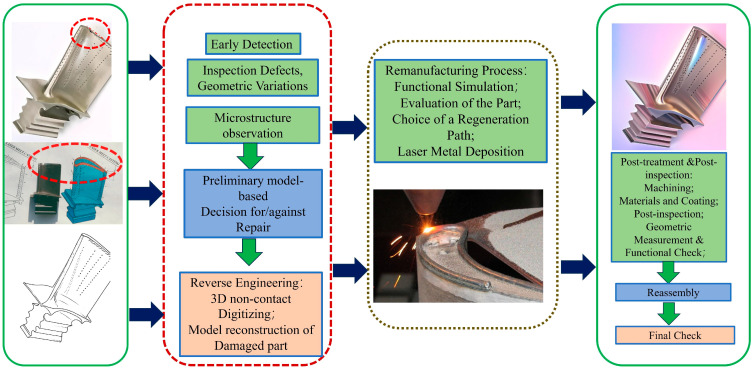
Macroscopic process flow of the turbine blade remanufacturing system implemented in this study, showing three main processes: from left to right: (1) the initial damage and (2) its initial assessment before remanufacturing process, (3) the laser-assisted material deposition, and (4) the precision finishing and inspection.

Modern remanufacturing processes based on reverse engineering (RE) technology typically involve multiple complex and sequential macro-level stages. The process entails initial inspection and disassembly, followed by reprocessing (e.g., laser metal deposition, Figure 2). Subsequent machining, which may involve heat treatment and surface modification (e.g., shot peening), is then conducted prior to reassembly and final testing. Figure 3 illustrates a flowchart of the remanufacturing process for damaged turbine blades.

By integrating these macro-level stages, the remanufacturing process restores damaged turbine blades and extends their service life, effectively giving them a second life.

In industrial remanufacturing systems, reverse engineering (RE) plays a critical role in reconstructing the 3D geometry of damaged components. The digitized model serves as a fundamental input for CAD/CAM-based toolpath generation. This enables an integrated additive–subtractive manufacturing approach, which combines laser-directed energy deposition (L-DED) for material restoration with precision five-axis CNC milling for final surface finishing. The toolpath generation process involves two main phases. The first phase uses three-axis additive deposition paths, which are derived from RE-generated surface compensation patches. The second phase involves five-axis subtractive machining trajectories, calculated according to final geometry requirements.

Post-processing algorithms then convert these toolpaths into machine-executable G-code. The accuracy of the RE-based geometry reconstruction is crucial for achieving final dimensional tolerances. This aspect is equally important as the deposition parameters, especially for complex turbomachinery components with the following characteristics:

(1) Aerodynamic surfaces require <50 μm form accuracy.
(2) Wall thickness variations must be controlled within ±0.1mm.
(3) Surface roughness Ra < 1.6 μm is typically specified.

### 3.2. Three-Dimensional Digitizing System

Turbine blades experience severe thermomechanical loading during service, resulting in progressive damage accumulation. As shown in Figure 1, the predominant failure modes include the following:

(1) Surface nicks and edge loss;
(2) Material erosion and wear;
(3) Crack initiation and propagation;
(4) Thermal–mechanical fatigue damage.

The remanufacturing of aerospace components, such as turbine blades, involves a significant hybrid manufacturing process. This process integrates automated inspection with both metal additive and subtractive techniques. Since turbine blades can exhibit various types of defects that differ from one part to another, it is critical to adapt the remanufacturing process to the specific geometrical degradation of each blade. Acquiring precise geometrical data is a fundamental step in this procedure. Such data enable accurate reconstruction of worn components.

**Figure 4:**
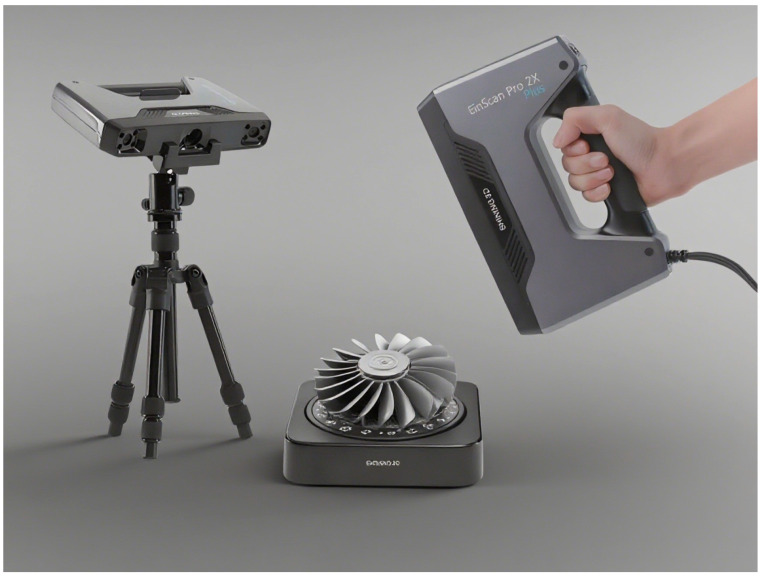
Turbine blade scanning process using a 3D laser scanner.

Here, a FARO laser scanner—a high-speed 3D laser scanning system—is employed for detailed measurement and digital archiving. Additionally, the EinScan Pro 2X 3D scanner (EinScan, Hangzhou, China) is used to rapidly acquire high-quality 3D models. This device supports 3D scanning of small to medium-sized physical objects with diverse detailing and accuracy requirements, effectively balancing scan detail with operational efficiency. Therefore, accurate 3D model reconstruction for blade remanufacturing requires a robust digital measurement system capable of providing high-fidelity geometric data. In this study, we selected a FARO laser scanner (shown in Figure 4) for digitizing worn blades based on a comprehensive evaluation of the following aspects:

(1) Component dimensional requirements;
(2) Measurement accuracy specifications;
(3) Scanning efficiency parameters;
(4) Data processing capabilities;
(5) Final reconstruction quality.

The complete turbine blade scanning methodology is illustrated in Figure 4. During its actual application, there are some drawbacks, as follows:

**(1) Limited Optical Line of Sight**

The intricate curvature and confined geometry near the blade root can obstruct the scanner’s optical path, resulting in partial or incomplete data acquisition. This limitation can introduce gaps in the point cloud, necessitating manual interpolation. This process may, in turn, compromise the accuracy of subsequent repair assessments.

**(2) Shadowing Effects**

Adjacent blade surfaces or the blade platform may occlude recessed regions, creating shadowed areas that are inaccessible to the laser beam. Consequently, the acquired scans often exhibit discontinuous patches, requiring repeated scans from alternate viewpoints or scanner repositioning. Such measures increase the inspection time and operational complexity.

**(3) Surface Reflectivity and Material Heterogeneity**

The blade root frequently features specialized coatings (e.g., thermal barrier coatings) or machining marks that induce non-uniform laser light scattering or absorption. These surface properties can generate excessive noise or geometric distortions in the point cloud, mandating post-processing operations to mitigate artifacts.

**(4) Scanner Resolution Limitations**

Conventional laser scanners may lack the spatial resolution required to resolve fine geometric features, such as cooling holes, filets, or localized erosion damage, within highly constrained regions. This deficiency raises the risk of undetected defects, potentially affecting the integrity of repair operations.

**(5) Environmental and Access Constraints**

In situ scanning applications (e.g., on-wing or in-engine inspections) often impose physical restrictions on scanner placement. Suboptimal positioning due to accessibility limitations can further degrade data quality and completeness.

Mitigation Strategies for Enhanced Data Acquisition.

To address these challenges, several strategies can be employed to improve scanning fidelity in blade root inspections.

Multi-angle scanning: Data acquisition from multiple orientations minimizes shadowing effects and improves coverage.

High-resolution scanning systems: Structured-light or precision laser scanners can enhance feature detection in complex geometries.

Controlled surface preparation: Temporary anti-reflective coatings (where permissible) may standardize reflectivity and reduce optical noise.

Multi-modal data fusion: Complementary techniques such as computed tomography (CT) or tactile probing can supplement laser scans in critical regions.

Advanced computational processing: Gap-filling algorithms (e.g., Poisson reconstruction) and noise-filtering methods can refine raw point cloud data.

The blade root region presents inherent inspection difficulties due to geometric constraints and material properties. While laser scanning remains efficient for most aerodynamic surfaces, the root zone often demands specialized approaches to ensure metrological accuracy. For mission-critical components, a hybrid inspection methodology integrating laser scanning with complementary non-destructive evaluation (NDE) techniques is recommended to achieve comprehensive defect characterization.

**Figure 5:**
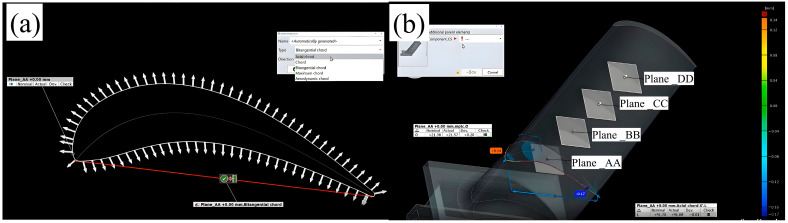
The scanning (**a**) and the slicing process (**b**) for the blade samples used in the laser metal deposition remanufacturing process.

The blade geometry was digitized using an EinScan Pro 2X 3D laser scanner (Figure 5a). The scanner achieved comprehensive surface capture, as shown in Figure 5b. With a specified accuracy of 0.04 mm in fixed scanning mode, the system successfully resolved fine blade features, which is demonstrated in the detailed reconstructed model. Key advantages of the scanner include the following:

(1) Rapid acquisition time (<5 min for full scan);
(2) High-resolution output (~2 million data points);
(3) Precise feature reproduction (sub-0.1mm fidelity).

The raw point cloud data were converted into a fine-resolution mesh and then imported into SolidWorks (https://www.solidworks.com/zh-hans/community/3dexperience-login, accessed on 1 February 2023) for parametric surface reconstruction.

The 3D laser scanning system completes the entire measurement process for a 0.7 m turbine blade in approximately 3 min. This includes both data acquisition and report generation. The process demonstrates a significant improvement in efficiency compared to conventional measurement methods.

The complex geometry and diverse defects of turbine blades make it challenging to obtain sufficient data in a single scan. Therefore, multiple scans from different orientations are essential. The resulting datasets are then integrated into a complete 3D model.

Accordingly, single-blade and three-blade components were scanned to obtain point cloud data. If available, at least one new component was also scanned for reference. The scanning results are presented in Figure 5.

During the retrieval of a nominal CAD model for worn components, the original technical drawings or related documentation may be incomplete or temporarily unavailable. When a nominal CAD model is absent, reverse engineering techniques must be applied to reconstruct a virtual repair surface model based on the geometric parameters of the damaged component surface.

The boundary of the defective region exhibits measurable geometric discontinuities. This is supported by the Gaussian curvature analysis of the point cloud data. The nonsmooth transition between intact and defective zones causes a sharp increase in Gaussian curvature at boundary points. This abrupt change enables the detection of defects via differential geometric analysis [32].

Typically, surface patches satisfy at least C^0^ continuity, but their borders align with curvature discontinuity lines. Several established computational methods are commonly used for curvature estimation in point cloud data. These include:

(1) Quadratic surface fitting [32];
(2) 3D Shepard surface interpolation [32];
(3) Implementation of the discrete Gauss–Bonnet theorem [32].

Among these, quadratic surface fitting achieves relatively higher accuracy in curvature estimation, especially when processing point clouds with noise. In this study, quadratic surface fitting is applied to estimate the curvature of each point in the substrate point cloud. Further details of this method are provided in reference [32].

### 3.3. Defect Treatment

The material is the ЖС6У alloy, developed in Russia; this is equivalent to the Chinese standard K465 superalloy—(9.5–11%) W, 8–9.5% Cr, (5.1–6%) Al, (1.5–2.5%) Ti, (9–10.5%) Co, (1.2–2.4%) Mo, (0.8–1.2%) Nb, ≤0.035% B, and ≤0.04% Zr. It is balanced using Ni. The Stellite X40 powder made by Kennametal Stellite™ (Shanghai, China) was used as the cladding layer.

**Figure 6:**
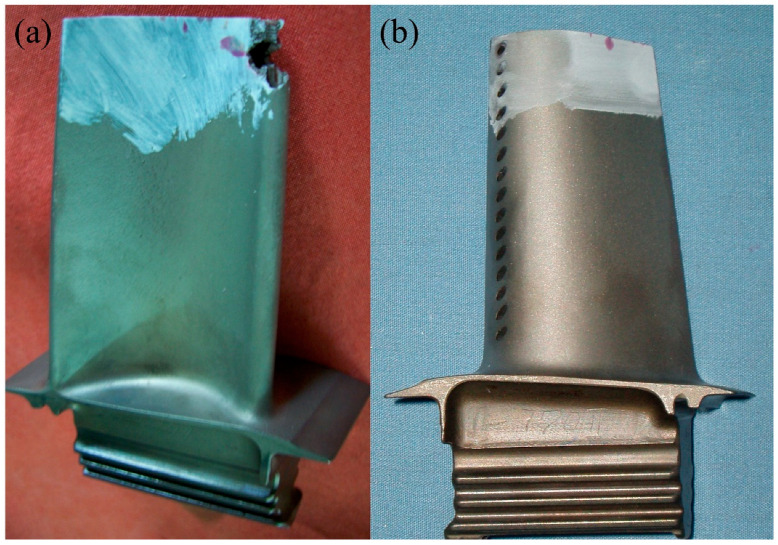

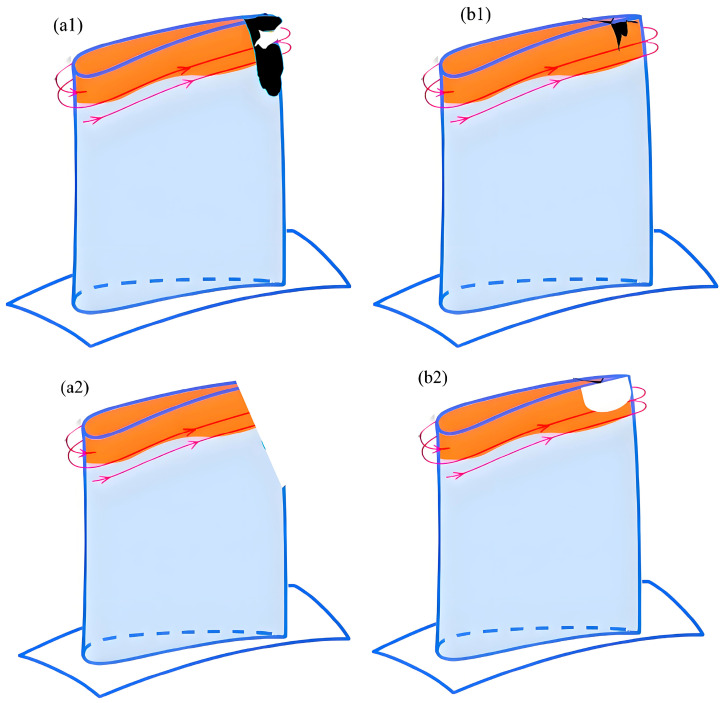
Damaged blades and their corresponding pre-remanufacturing base contour. Samples of the damaged blades (**a**,**b**), the schematic diagram of defect extraction (**a1**,**b1**), and the simplified defect model before remanufacturing (**a2**,**b2**).

The experimental setup utilized a 1 kW fiber laser system integrated with a coaxial powder feeding nozzle. High-purity argon (99.99%, Air Liquide) was employed for two purposes: as a powder carrier gas (flow rate: 3–5 L/min) and as a shielding gas to prevent oxidation of the molten pool (flow rate: 10–15 L/min). The scan speed was set between 5 and 20 mm/s, and the overlap ratio ranged from 30% to 50%. As shown earlier in Figure 3, the experimental setup used in this study was also employed to fabricate all samples. The laser power was selected according to the blade substrate material. Two types of artificial defects were created on the blades; their initial appearances are shown in Figure 6a,b. To support laser metal deposition planning, the geometry of each defect was digitally extracted. The extracted geometry was then simplified to facilitate the laser remanufacturing process, as illustrated in Figure 6a2,b2. The scanning height was automatically adjusted based on the simplified model of the damage location.

As shown earlier in Figure 3, the same setup also produced all samples in this study. The laser power was adjusted based on the blade substrate material.

Two types of artificial defects were created on the blades; their as-made morphologies are shown in Figure 6a,b. To aid in laser metal deposition planning, each defect geometry was digitally extracted. The extracted geometry was then simplified to facilitate the laser remanufacturing process, as shown in Figure 6a2,b2. The scanning height was automatically adjusted according to the simplified model of the damage location.

### 3.4. Parameter Selection

However, the fabrication of high-quality coatings on the blade via Laser Metal Deposition confronts several challenges. These requirements are twofold. First, suitable high-performance materials with excellent laser processability must be selected. Second, the underlying mechanisms for mechanical property anisotropy must be elucidated to guide its effective mitigation. Furthermore, precise control over the geometrical morphology, which is critically dependent on process parameters, remains a key issue for dimensional accuracy. And a fundamental challenge in laser metal deposition is to unravel the complex coupling between thermal, hydrodynamic, and metallurgical processes. Current knowledge is insufficient to describe the integrated heat-flow-grain evolution across multiple scales. A particular challenge is understanding the synergistic effects that emerge from the process’s inherent non-isothermal nature. The traditional parameter development for LMD of a novel alloy entails a three-stage approach [33]. It begins with single-track deposition to down-select the processing window, then proceeds to single-layer cladding for morphological optimization, and finally fabricates multi-layer bulk components for performance evaluation.

During the laser repair process of Ni-based superalloy turbine blades, different process parameters, such as scanning path, laser power, scanning speed, and heat treatment, significantly affect the final microstructure and properties.

While the accuracy, adaptability, and relevance of the above-mentioned models have been verified, existing research has predominantly centered on the influence of process parameters on forming quality. Nevertheless, the forming quality exhibits high sensitivity to minor modifications in these parameters, often leading to pronounced variations. Consequently, systematic sensitivity analysis is imperative for providing effective guidance in process parameter optimization. Cai et al. recently reported a study aiming to elucidate the coupling mechanism between the thermal field, fluid flow, and grain microstructure evolution in laser cladding [34]. Combined experimental and numerical simulations were employed to investigate the molten pool’s thermal behavior, flow dynamics, and solidification parameters. Quantitative relationships were established among solidification parameters, conditions, and resultant microstructures. This enabled an effective simulation of the microstructural evolution and clarified the mechanism behind the columnar-to-equiaxed transition (CET).

**Figure 7:**
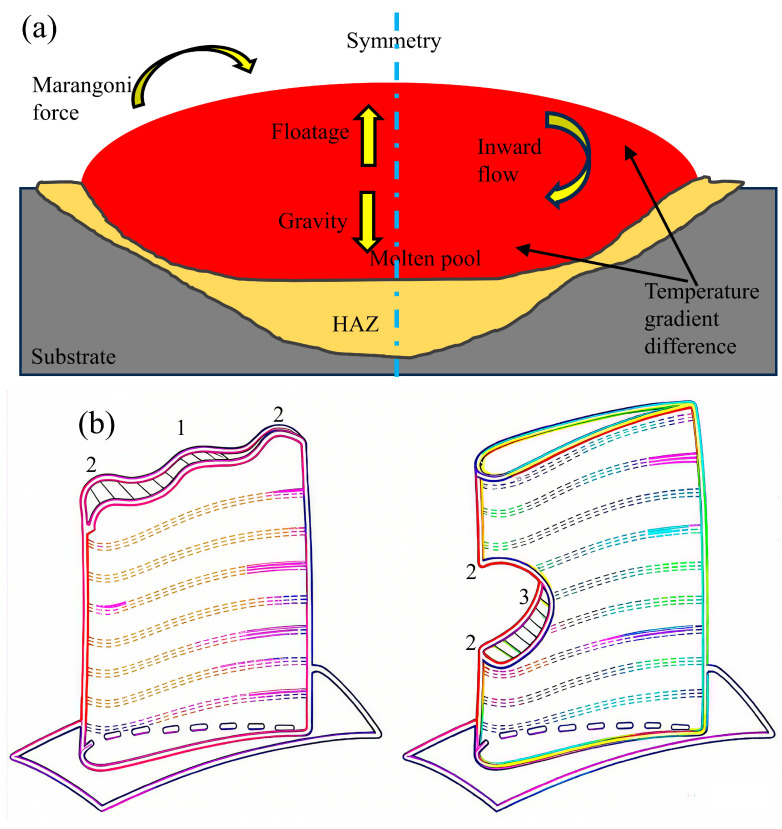

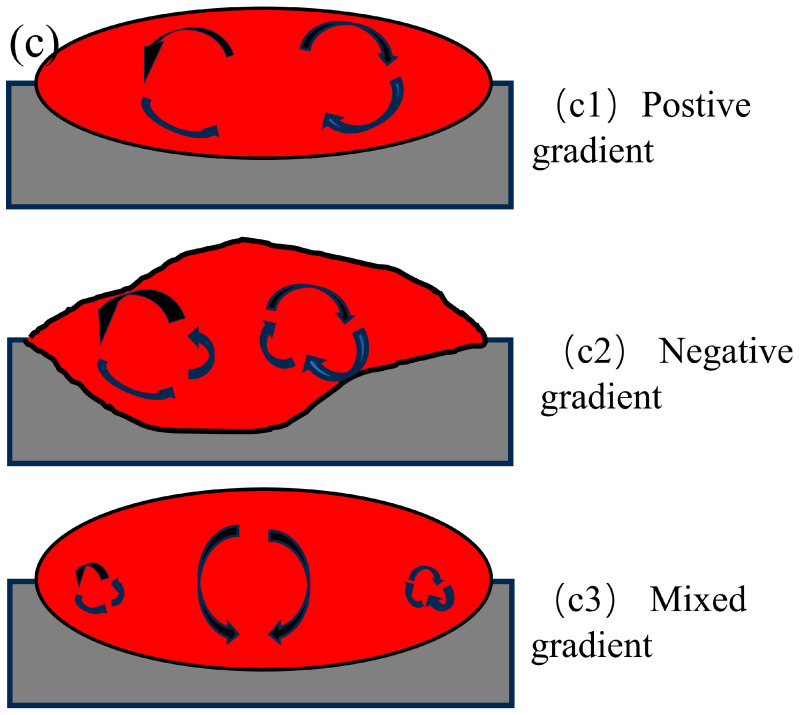
(**a**) Schematic diagram of standard driving force in laser metal deposition process; (**b**) defect location formed on the blade before laser coating mainly have two different situations, i.e., on the top blade and on the blade side; (**c**) flow patterns and molten pool shapes produced by different rates temperature change in surface tension coefficient, i.e., positive gradient; negative gradient and mixed gradient. (here, position 1 corresponding to situation Figure c-c1, and position 2 corresponding to situation Figure c-c2, position 3 corresponding to Figure c-c3).

On the surface of the molten pool, the uneven distribution of the temperature gradient drives circulation within the pool through Marangoni forces. Marangoni convection exerts a decisive influence on the morphological development of the molten pool. The surface tension coefficient is a primary driver of liquid metal flow in the molten pool. The sign of the surface tension temperature coefficient dictates the flow direction of the non-isothermal circulation in the cross-section, shifting from inward to outward as it changes from positive to negative [34]. As depicted in Figure 7a, the resultant molten pool exhibits a broad, shallow profile with a convex surface contour.

As shown in Figure 7a, the flow of molten pool in the laser cladding is affected by multiple factors [34]. Consequently, a thorough understanding of the molten pool flow-thermal coupling is crucial for optimizing process parameters and ensuring quality outcomes. The formation, stability, and morphology of the melt pool are intricately linked to the coupled thermal and velocity fields during laser cladding. Marangoni convection governs the fluid flow and thermal transport within the melt pool, thereby establishing the foundational basis for predicting elemental homogenization and solidification parameters.

The thermocapillary gradient coefficient (η) varies with the substrate location, as illustrated in Figure 7b. This variation dictates the magnitude and direction of the Marangoni force on the molten pool surface, thereby inducing distinct flow patterns. Lee and Farson [35,36] investigated how the thermocapillary gradient coefficient (η) influences Marangoni force and molten pool flow. Their findings indicate that when η is negative, the Marangoni force points from high to low-temperature regions. This force drives a radially outward flow in the molten pool, as shown in Figure 7c1. When the coefficient is positive (η > 0), the resulting force is directed from low- to high-temperature regions. This establishes a convergent inward flow within the molten pool, as shown in Figure 7c2. Under a hybrid thermocapillary gradient, the molten pool develops a highly complex flow regime. This complexity manifests as an undulating, wavy morphology at the pool bottom (Figure 7c3). Furthermore, studies on inclined substrates show that changes in gravitational orientation disrupt pool stability and degrade its geometry and flow [34,36]. These effects markedly reduce forming precision and quality. Consequently, molten pool behavior directly governs the formability, microstructure, and mechanical properties of clad components. Also, the thermocapillary gradient coefficients are quite different and complex in multi-pass and multi-layer cladding. Then the selected processing parameters should be chosen by different conditions.

**Figure 8:**
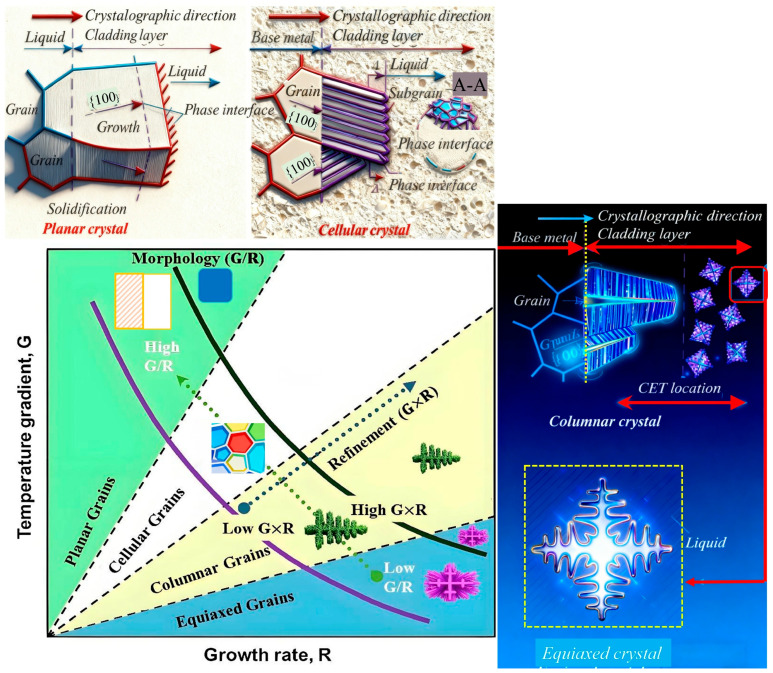
Effect of Temperature gradient (G) and solidification rate (R) on Grain morphology and size map: G vs. R, adapted from S Kou [38].

As delineated in Figure 8, the cooling rate (G × R) and the temperature gradient-to-solidification rate ratio (G/R) are critical factors governing the morphological evolution and scale of solidification microstructures [37,38]. An increased cooling rate promotes microstructural refinement, whereas the G/R ratio dictates the morphological transition from planar to cellular, columnar dendritic, and ultimately equiaxed dendritic structures [37,38]. So, the selected processing parameters are quite difficult to achieve, and one should consider many factors.

The introduction of new geometries, process parameters, and materials in Additive Manufacturing is often a time-consuming and costly endeavor. This is particularly true for Laser-Directed Energy Deposition (DED-L), where the highly dynamic and non-equilibrium nature of the melt pool necessitates iterative experimental campaigns to characterize and quantify process behavior. The considerable expense associated with these tests consequently elevates overall manufacturing costs. Therefore, a Digital Twin (DT) for the DED-L process presents a substantial value proposition by effectively minimizing the dependency on extensive experimental testing [39]. Some researchers have employed a Digital Twin of the process to help mitigate these extensive trial-and-error efforts. The Digital Twin (DT) was experimentally validated using an industrial-grade Directed Energy Deposition (DED-L) system equipped with in situ process monitoring. In all cases, the digital twin (DT) agreed well with experimental data and metallographic inspections [39]. This validation was achieved at a reasonable computational cost.

Although the confluence of Digital Twins and machine learning offers a pathway to proactive manufacturing control, the critical challenge of executing real-time, prediction-driven optimization for highly nonlinear systems remains. Chen et al.’s study tackles this challenge by introducing a Model Predictive Control (MPC) framework, validated within a Directed Energy Deposition (DED) additive manufacturing process [40]. The results demonstrate that the proposed MPC successfully tracks melt pool temperature for quality assurance. By regulating laser power, it also confines the melt pool depth, thereby suppressing porosity formation [38]. The proposed MPC framework outperforms conventional PID control by generating markedly smoother laser power profiles with reduced fluctuation [40]. It simultaneously achieves comparable or superior regulation of the melt pool temperature. This performance superiority validates the MPC’s proactive control capabilities, which are underpinned by its inherent use of time-series prediction and real-time optimization. These attributes position MPC as a pivotal tool for advancing Digital Twin implementations and enabling robust real-time process optimization in manufacturing [40].

### 3.5. In Situ Monitoring

Laser Metal Deposition (LMD)remanufacturing is a prominent metal additive manufacturing technique employed in remanufacturing and the direct fabrication of complex components, such as superalloy blades. The LMD process involves complex multi-parameter interactions and rapid thermophysical transients during melting and solidification. These transients can induce microstructural defects such as porosity, solidification cracking, and residual stress-induced deformation. These inherent defects, coupled with challenges in process repeatability and operational reliability, significantly constrain the broader application of laser cladding technology. Consequently, mitigating defect formation, enhancing process stability, and achieving precise control over the cladding process represent critical challenges that must be addressed in ongoing LMD research.

**Figure 9:**
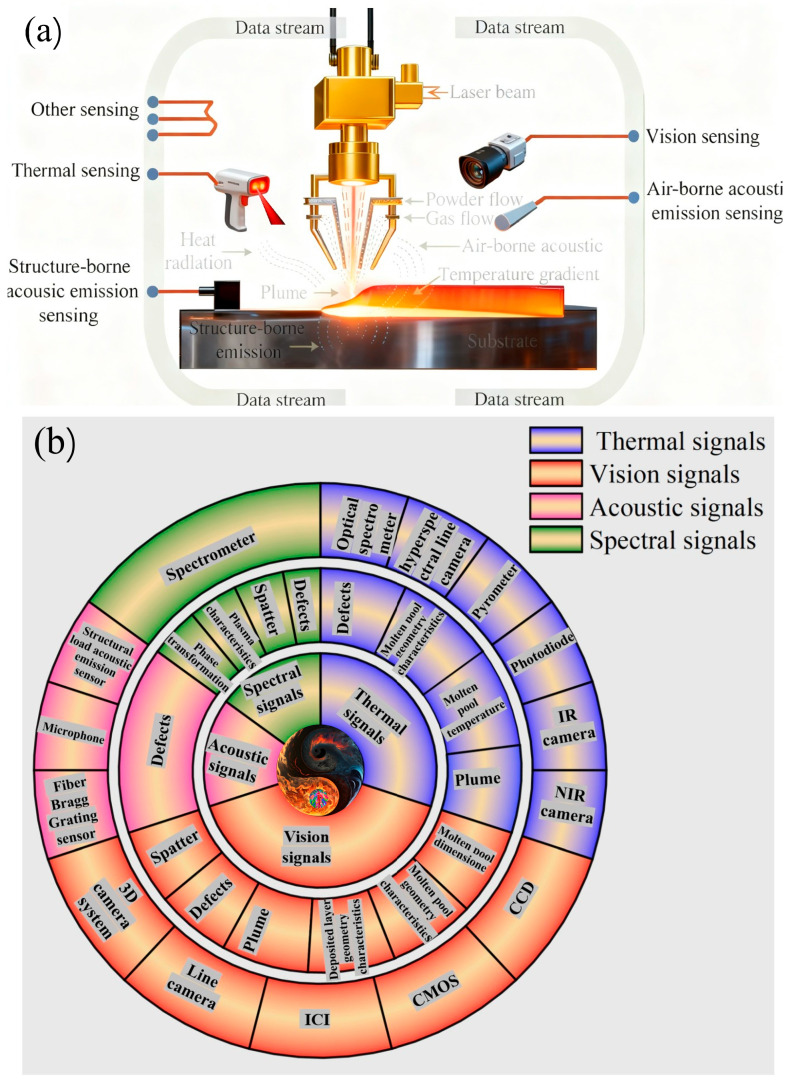
(**a**) Schematic diagram of the generation and propagation of physical signals in laser metal deposition remanufacturing process. (**b**) Radial Map of In Situ Monitoring Technologies used for defect detection and laser metal deposition remanufacturing. The inner circle represents the signal type to be monitored, and the outer circle shows the techniques implemented to monitor the process.

In laser cladding, the molten pool constitutes the fundamental physical domain. Its dynamics—encompassing formation, evolution, and solidification—directly manifest the complex metallurgical reactions occurring during deposition. The spatiotemporal characteristics of the molten pool act not only as highly sensitive indicators of variations in process parameters but also as critical determinants governing the final microstructure and performance of the clad layer. Consequently, the development of advanced online monitoring techniques coupled with real-time control strategies for molten pool dynamics has emerged as a pivotal research direction aimed at enhancing process stability and ensuring product reliability [41,42,43,44]. And Figure 9 shows the (a) Schematic diagram of the generation and propagation of physical signals in laser metal deposition remanufacturing; (b) Radial Map of In Situ Monitoring Technologies used in for defect detection in laser metal deposition remanufacturing. The inner circle represents the signal type to be monitored, and the outer circle shows the techniques implemented to monitor the process.

The acquisition of molten pool images is fundamental for characterizing its morphology and thermal distribution. Such data enable the establishment of critical correlations among process parameters, molten pool characteristics, and the resulting cladding layer properties. By actively adjusting process parameters based on these correlations, it becomes feasible to regulate molten pool behavior and ultimately control the quality of the deposited layer, thereby enhancing process reliability. Given its pivotal role in quality assurance, research on monitoring and control methodologies for laser metal deposition remanufacturing has attracted considerable scholarly attention in recent years.

The study performed by Mingzhang Chen et al. examined the mechanisms of cracking and corresponding suppression strategies in directed energy deposited IN738 superalloy, employing microstructural characterization and thermodynamic calculations [43]. It elucidated the underlying causes of crack formation and effective mitigation approaches, thereby providing valuable technical insights for the additive manufacturing of nickel-based superalloys.

In laser metal deposition remanufacturing, thin-walled sections often exhibit surface waviness (humping). This results in three major drawbacks: elevated residual stress, increased crack susceptibility, and reduced fatigue life. The crack formation mechanism remains elusive due to a lack of adequate operando monitoring methods [44]. Tristan G. Fleming et al. developed a multi-modal monitoring approach that integrates inline coherent imaging (ICI) for in situ surface topology and crack detection. This was complemented by synchrotron X-ray imaging for sub-surface analysis of crack healing and growth [44]. The system achieved a registration accuracy of under 10 µm laterally and 3 µm in depth between ICI and the laser position. Furthermore, ICI surface data showed strong agreement with X-ray radiographs (correlation > 0.93) [44]. Critically, in humped CM247LC thin-walls, ICI detected in situ crack openings as small as 7 µm and sub-surface signals, directly linking surface waviness to crack initiation [44].

### 3.6. Digital Twin and Model Predictive Control (MPC)

The introduction of new geometries, process parameters, and materials in Additive Manufacturing is often a time-consuming and costly endeavor. This is particularly true for Laser-Directed Energy Deposition (DED-L) in remanufacturing. The highly dynamic, non-equilibrium melt pool necessitates iterative experiments to characterize process behavior. The considerable expense associated with these tests consequently elevates overall manufacturing costs. Therefore, a Digital Twin (DT) for the DED-L process presents a substantial value proposition by effectively minimizing the dependency on extensive experimental testing [45]. As stated, some researchers have employed a Digital Twin of the process to help mitigate these extensive trial-and-error efforts. The Digital Twin (DT) was experimentally validated using an industrial-grade Directed Energy Deposition (DED-L) system equipped with in situ process monitoring. And the results confirmed that in all cases, the DT shows a high resemblance with the experimental data and metallographic inspections at a reasonable computational cost [45].

Although the confluence of Digital Twins and machine learning offers a pathway to proactive manufacturing control, the critical challenge of executing real-time, prediction-driven optimization for highly nonlinear systems remains. Chen et al.’s study tackles this challenge by introducing a Model Predictive Control (MPC) framework, validated within a Directed Energy Deposition (DED) additive manufacturing process [40]. Their results demonstrate that the proposed MPC successfully achieves precise melt pool temperature tracking for quality assurance, while its regulatory action on laser power effectively confines melt pool depth, thereby suppressing porosity formation [40]. In comparison to a conventional Proportional–Integral–Derivative (PID) controller, the proposed MPC framework generates laser power profiles that are markedly smoother and exhibit reduced fluctuation, while simultaneously delivering comparable or superior melt pool temperature regulation [40]. This performance superiority validates the MPC’s proactive control capabilities, which are underpinned by its inherent use of time-series prediction and real-time optimization. These attributes position MPC as a pivotal tool for advancing Digital Twin implementations and enabling robust real-time process optimization in manufacturing [40].

### 3.7. Remanufacturing Protocols

Jens Aschenbruck et al. reported that the shape damage is the most common defect, and it primarily appears on the edge and tip of turbine blades [46,47]. Although gas tungsten arc welding (GTAW) is widely used for repairing aerospace components due to its low cost and efficiency [8,9], it has significant limitations. The process relies on a high-heat electric arc, creating a substantial heat-affected zone (HAZ). This often leads to defects such as liquation cracking, dilution, and component distortion. These challenges are exacerbated in high-temperature alloys containing high levels of Al and Ti [48]. The thermal input from arc welding and subsequent PWHT promotes hot cracking and extensive strain-age cracking in the HAZ, which can inflict secondary damage on the repaired component.

**Figure 10:**
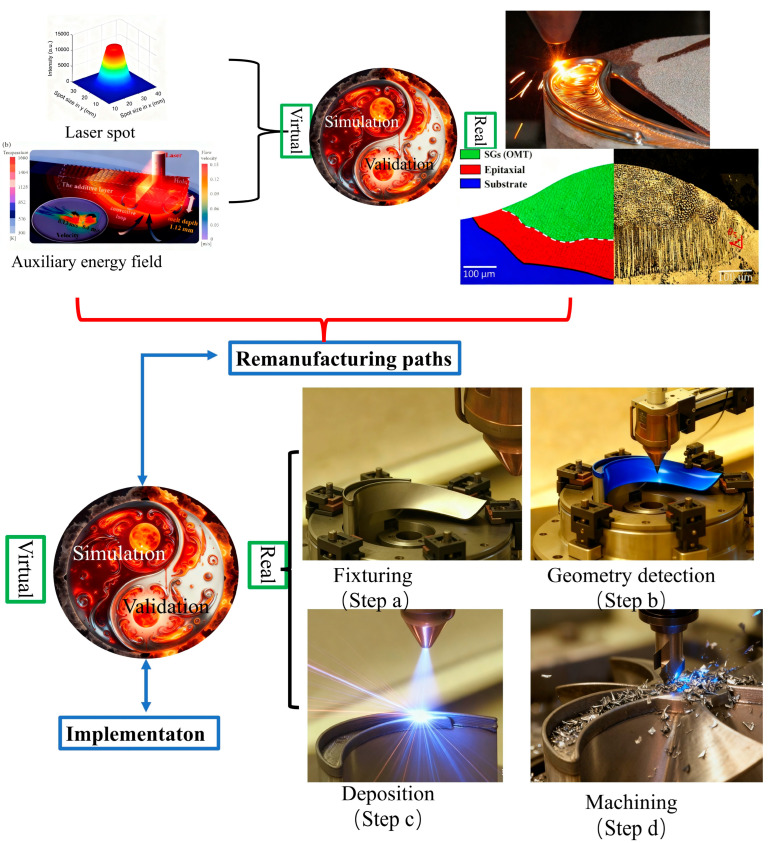
Development of regeneration steps for the turbine blade repair between simulation and experiment [47].

In the LMD process, repair material in the form of powder or wire is coaxially delivered into the defect region. This enables multi-layer, path-guided filling and reconstruction of the damaged area. The process is characterized by high heating/cooling rates and low dilution, promoting the formation of a sound metallurgical bond. Furthermore, components repaired via LMD demonstrate substantially reduced thermo-mechanical deformation, such as warpage and distortion, compared to those repaired by arc welding. This reduction in deformation minimizes the need for subsequent post-processing, thereby decreasing both the time and cost required to restore dimensional accuracy and service performance. And flow chart is illustrated in Figure 10 [47].

Nevertheless, a significant challenge remains in balancing precision with efficiency. Specifically, the process must prevent both excessive material deposition and the introduction of secondary defects during repair. A paramount objective in repair is to restore geometry while mitigating over-deposition, thereby minimizing the investment in subsequent post-processing. To this end, diverse in situ sensors are employed to monitor the process, enabling the prediction and control of repair outcomes. Extensive research in this domain has demonstrated promising results, affirming the efficacy of sensor-based strategies for process optimization.

Penaranda et al. [49] pioneered a geometry-based adaptive laser cladding method that dynamically modulates laser power in response to local blade thickness, thereby enabling precise control over the cladding process. Their investigation further revealed that the relationship between clad width and machined thin-wall width evolves through three distinct phases with increasing laser power.

For turbine blade repair, we investigate Gradient Laser Power (GLP) deposition. Unlike constant laser power methods, GLP progressively reduces laser power with increasing deposition height [50]. Their results empirically demonstrate that this dynamic power adjustment inherent to GLP significantly enhances both cladding quality and dimensional accuracy. This approach thereby offers a novel and efficient solution for the dimensional restoration of geometrically complex components like turbine blades.

It is widely documented that over 90% of turbine blade failures are predominantly attributable to cracks induced by the synergistic effects of fatigue and creep. In addressing this issue, brazing has emerged as the predominant technique for the surface repair of these critical components. This process utilizes filler metals with a melting point lower than that of the base material. A distinct advantage of brazing lies in the uniform heating it facilitates, which effectively mitigates thermal stress concentration and minimizes the risk of dimensional deviations. Furthermore, compared to conventional welding, brazing induces minimal microstructural alterations in the base metal. The resulting high-quality metallurgical bond ensures the repaired component retains excellent mechanical properties, thereby enabling reliable performance in demanding service environments [47].

Recent advances in brazing technology have yielded several promising repair methodologies with significant potential for application in the restoration of aeroengine turbine blades, particularly in addressing surface cracks. Among these, Transient Liquid Phase (TLP) bonding and Wide-Gap Brazing (WGB), laser brazing, and Hybrid Brazing represent the four most prominent techniques. The integrity of the brazed repair is critically dependent on the cleanliness of the substrate surface prior to reconstruction. Within the blade repair process, two substantial challenges persist: the effective removal of thermal barrier coatings (TBCs) and the complete elimination of oxides from narrow, deep cracks. In WGB, excessive gap width under non-pressurized conditions has been observed to result in an increased volume fraction of voids. Furthermore, the selection of optimal filler materials, additives, and their respective compositions for different material systems requires extensive validation through experimental data [47].

Laser brazing produces highly localized, non-uniform heating—unlike furnace brazing’s uniform thermal distribution. Its heat input and energy density vary significantly with distance from the beam center. This process generates substantial thermal gradients, with over 300 °C difference between the top and bottom of the joint. Such gradients increase the risk of stress concentration and solidification cracking [47]. Current research efforts are predominantly directed toward the laser brazing of high-temperature alloys. Given that turbine blades are typically fabricated from these very materials and are susceptible to in-service damage such as fatigue cracks, laser brazing presents considerable application prospects for their repair [47].

Although hybrid brazing techniques demonstrate superior repair performance, they currently fall short of effectively restoring the single-crystal (SC) microstructure essential for SC blade substrates. Furthermore, the suitability of the repaired surface morphology and resultant microstructure for subsequent processing steps—particularly the deposition of thermal barrier coatings (TBCs) that confer enhanced resistance to wear, oxidation, and Ca-Mg-Al-Si silicate (CMAS) corrosion—remains an area requiring extensive further investigation [47].

## 4. Microstructure and Mechanical Properties

### 4.1. Microstructure

**Figure 11:**
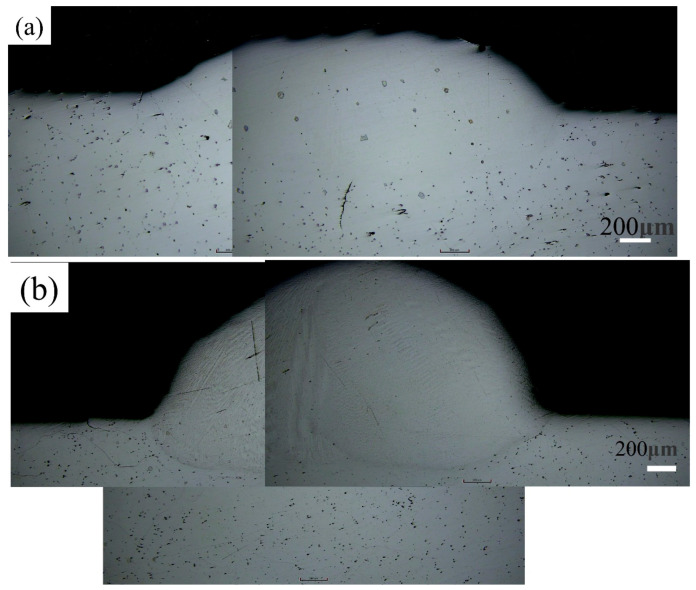
Microscope images of the cross-section of the laser metal remanufacturing deposition: a sample of a damaged turbine blade (**a**) lower height coating and (**b**) higher height coating under different processing parameters.

The microstructure of the laser additive-manufactured (LAM) layer, observed using scanning electron microscopy (SEM), is shown in Figure 9. The grains grow in a layer-by-layer manner. A fine-grained zone is present in the middle of each layer, while coarse-grained structures form at the interlayer junctions. Figure 11 shows the optical microscopy (OM) image of the LAM-formed layer, which also exhibits a layered grain growth pattern. Similarly, fine grains are located in the central part of each layer, and coarse grains appear at the layer interfaces. During the deposition of the coating on the damaged superalloy turbine blade, the Marangoni effect promotes thorough mixing and interdiffusion between the molten coating and the melted substrate. This results in a well-bonded metallurgical interface after solidification, as shown in Figure 11a,b. Moreover, the coating-substrate interface is free from microcracks and pores. This can be mainly attributed to the optimized scanning strategy and processing parameters used in the laser metal additive remanufacturing process, which help reduce interfacial and coating defects.

The sample was examined at 100× magnification. The largest pore diameter is less than 4 µm, and the porosity is below 2%.

**Figure 12:**
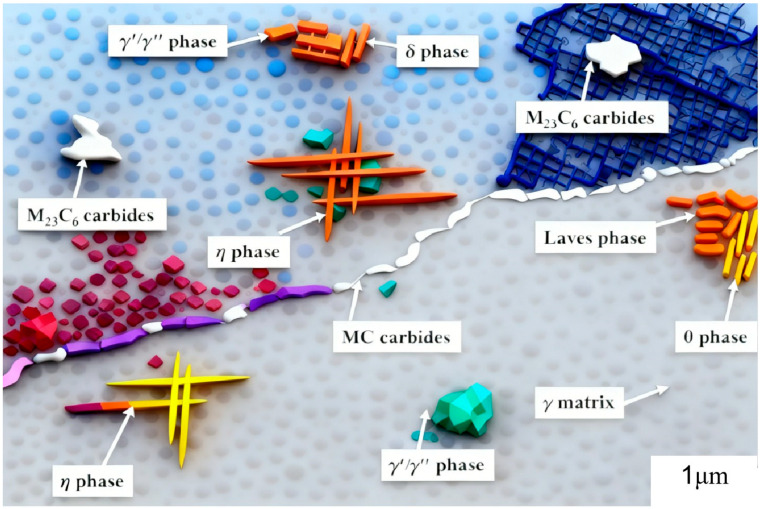
Schematic representation of microstructure of Inconel superalloys, depicting the different phases formed [28].

Ni-based superalloys are typically composed of over ten alloying elements. In this multicomponent system, nickel (Ni) is the primary constituent, forming the face-centered cubic γ matrix. Chromium (Cr) is the second most abundant element, while other additions facilitate the formation of various strengthening precipitates and secondary phases. The addition of Al, Ti, and Ta enables the precipitation of ordered, coherent γ′ (Ni_3_(Al, Ti)) phase, which serves as the primary strengthening mechanism. The elevated temperature strength of these superalloys is principally governed by the morphology, size, and volume fraction of these γ′ precipitates. The microstructure is further complemented by secondary phases. These comprise metal carbides, a needle/plate-like orthorhombic δ phase (Ni_3_(Nb, Ti)), and topologically close-packed hexagonal Laves phases (e.g., (Ni, Fe, Cr)_2_(Nb, Mo, Ti)). The high-temperature mechanical properties of IN-series superalloys are collectively governed by their alloying additions, resultant microstructure, and distribution of constituent phases. A schematic representation summarizing these characteristic microstructural features and the corresponding phases is provided in Figure 12 [28].

As mentioned in Figure 12, there exists many forms of cascade failure progression in a gas turbine system initiated by blade integrity compromise. Given the stringent manufacturing and control protocols governing turbine blade utilization, the present work mainly focuses specifically on creep-related phenomena. Creep deformation of superalloys is presented that is sensitive to chemical composition, surface condition and microstructure. Their study (Jiawang Chen et al. [51]) systematically examined the thermal fatigue behavior and accompanying microstructural evolution of a novel superalloy ZGH451 fabricated by directed energy deposition (DED), at temperatures of 900, 1000, and 1100 °C. The investigation placed particular emphasis on the mechanisms of crack initiation and propagation. The principal findings are summarized as follows. The as-fabricated ZGH451 superalloy exhibited a characteristic microstructure comprising a homogeneous γ matrix, γ’ precipitates (∼300 nm in size) serving as the strengthening phase, and a limited population of eutectic constituents. Under thermal fatigue loading, cracks preferentially nucleated at defects near the notch root. The crack propagation direction, observed at approximately 45° to the building direction (BD), is indicative of shear-driven failure along the {111}‹110› slip systems. Crack initiation was primarily driven by cyclic thermal stresses, with both the initiation life and propagation rate showing a strong dependence on the applied peak temperature. The crack propagation behavior was temperature-dependent. At 1000 and 1100 °C, the crack growth rate exhibited a characteristic transient stage, following a sequence of initiation, acceleration, and deceleration. In contrast, at 900 °C, the cracks displayed only accelerated growth, a behavior attributed to minimal oxidation effects resulting from the alloy’s superior oxidation resistance at this temperature. The deformation of the γ’ phase was primarily governed by thermally induced stresses. Concurrently, oxidative degradation progressively compromised the mechanical integrity of the γ/γ’ microstructure adjacent to the crack path, resulting in crack tip blunting with an increasing number of thermal cycles. Consequently, the damage evolution during thermal fatigue was attributed to the synergistic interaction between cyclic plasticity driven by thermal stress and environment-assisted microstructural degradation.

### 4.2. Mechanical Properties

**Figure 13:**
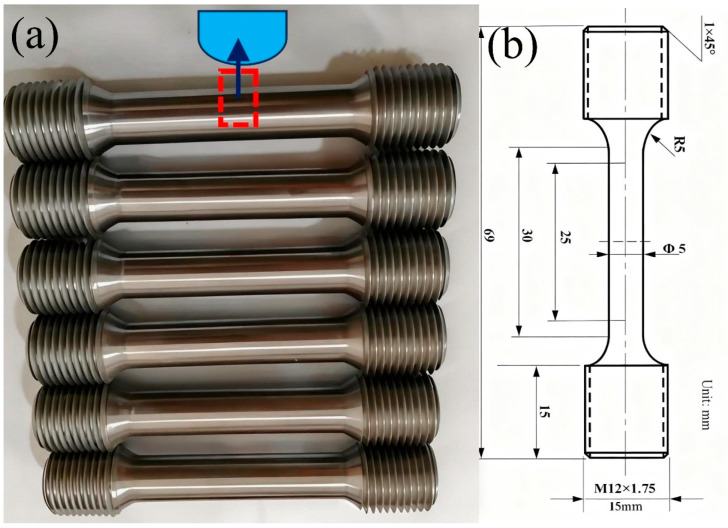
Standard tensile test specimens with a U-notch machined in the middle section (**a**) and their dimensions (**b**).

Independent standard tensile test specimens were used, with a U-notch being machined in the middle section for laser remanufacturing welding. Figure 13 shows the mechanical test samples and the sketch illustration of the U-shaped groove that is formed in the middle of the mechanical test sample. Then, mechanical property testing was conducted to simulate the repaired blade sample. Mechanical tests were performed according to key international standards for mechanical testing of aerospace superalloys, such as GE Aviation S1000: Proprietary superalloy testing specifications; SAE AMS2750H-2024 [52] (Pyrometry for high-temperature testing) and ASTM E606-92 [53].

The coating layer has a hardness of about 40–46 HRC, while the K 465 substrate is about 36–39 HRC. Creep resistance was evaluated at 850 °C under atmospheric conditions for both base metal and weld-repaired specimens. To isolate the weld-affected zone for testing, specimens were fabricated with a 1.6 mm diameter central bore. This design effectively removed the base material core, creating a true cross-weld testing configuration. Applied stress levels ranged from 150 to 300 MPa, with test durations extending to 5000 h for base metal and 3570 h for repaired specimens. The material’s behavior under creep-fatigue interaction was assessed using low-cycle fatigue (LCF) tests, encompassing both continuous-cycle (LCF-CC) and tensile-hold (LCF+HT) conditions. And the Ultimate Tensile Strength under different temperature for cast and heat treatment sample can be seen in the supplement file Table S1.

At 850 °C, the tensile properties of the laser repair welds reached 85–90% of the base material’s values. Fracture analysis revealed ductile failure within the gauge section, with most failures propagating through the weld and heat-affected zone (HAZ). Notably, one specimen fractured in the parent material. The LCF results, with or without a tensile-hold cycle, were remarkably consistent.

Furthermore, the elastic modulus values of the laser metal additive remanufactured gas turbine blade, the original blade, and the coating–substrate interface were found to be comparable.

**Figure 14:**
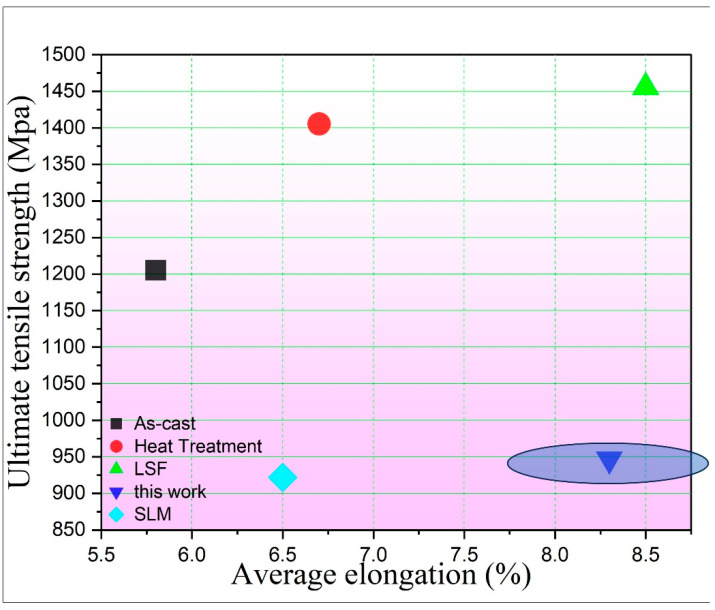
Comparison of average tensile strength and elongation for K465 alloy fabricated by different manufacturing processes. (The data for as cast, heat treatment, and LSF is from ref. [54], while SLM is from ref. [55]. LSF-laser solid formed, SLM-selective laser melting.)

A comparative assessment of the mechanical properties achieved in this work with existing literature is presented in Figure 14. The UTS -elongation profile of the K 465 alloy under the current HT-A protocol is systematically compared against a corpus of published data derived from various manufacturing processes. This study compares AM specimens (via LSF [54] and SLM [55,56], including heat-treated [55]) with cast counterparts [54]. All were subjected to HT-A, illustrating the relative performance of the present material.

As presented in Figure 14, the LSFed (laser solid formed) K465 superalloy in the as-deposited condition exhibits superior room temperature tensile properties compared to its as-cast and heat-treated counterparts [39]. It demonstrates an average ultimate tensile strength of 1205.4 MPa, surpassing the as-cast and heat-treated conditions by approximately 250 MPa and 200 MPa, respectively. Furthermore, its yield strength and elongation are measured at 917.4 MPa and 8.5%, representing the highest values among all the conditions evaluated [54]. And in our work, the tensile strength is 947 ± 21 MPa and the elongation is 8.3 ± 0.7%. The comparative results in the literature with our work are illustrated in Figure 14. While the UTS for SLM is 922 MPa, and the elongation is 6.5% [55,56].

## 5. Post-Treatment

Beyond the optimization of processing parameters, the implementation of a systematic post-processing strategy through heat treatment offers a viable pathway to enhance the performance of additively manufactured nickel-based superalloys. The application of appropriate thermal cycles promotes a more homogeneous microstructure, which consequently mitigates anisotropic mechanical properties, including tensile strength and elastic modulus [57]. Furthermore, tailored heat treatments can effectively suppress undesirable phase transformations that are otherwise driven by micro-segregation and residual strains, thereby stabilizing the microstructure.

For example, a novel three-stage heat treatment protocol was engineered specifically for IN625-on-Rene125 turbine blade repair. This methodology aims to achieve three key objectives: First, dissolve secondary phases at 1220 °C. Second, suppress brittle constituent precipitation by controlled air-cooling to 590 °C. Finally, promote interfacial stress relaxation via dislocation recovery during a final slow-cooling stage. This study systematically examines the laser deposition of IN625 alloy onto cast René125 superalloy substrates. A central challenge in such dissimilar material combinations involves controlling the interfacial microstructure. This requires eliminating deleterious brittle phases and mitigating residual stresses. To address this, a tailored multi-stage heat treatment cycle was developed and optimized. The results demonstrate that this precisely engineered post-deposition thermal cycle significantly enhances the metallurgical bond integrity at the interface. This approach extended the fatigue life of the superalloy by up to 3.96 times. It thus provides a strategic framework for optimizing additive manufacturing in high-temperature applications [57].

The interplay among annealing temperature, residual stress, and recrystallization behavior in DED-repaired DD32 single-crystal superalloy was systematically elucidated. The stress relief mechanism was identified to transition with increasing temperature, encompassing stress relaxation, plastic deformation, creep, and finally recrystallization. At the microscopic level, stress relief is primarily governed by the slip and subsequent annihilation of dislocations. An optimal annealing window was established at 800 °C. This temperature effectively promotes dislocation-mediated stress relief while suppressing competitive recrystallization, thereby preserving single-crystal integrity [58].

Process-induced defects, particularly those at or near the surface, are among the most detrimental factors to the fatigue performance of additively manufactured (AM) metal alloys. Among these, porosity and voids represent the most prevalent type of volumetric defect, which act as preferential sites for crack initiation under cyclic loading. Furthermore, the severity of their impact is well-established to depend on key defect characteristics. These include size, shape, morphology, and, most critically, proximity to the free surface, which collectively determine the resultant fatigue properties. Hot isostatic pressing (HIP) is widely regarded as an effective post-processing technique for enhancing the fatigue behavior of additively manufactured (AM) metal alloys. However, its efficacy is highly material-dependent. For AM IN625 alloys, HIP treatment demonstrates an insignificant effect on fatigue performance, irrespective of prior heat treatments; the fatigue response in the HIPed condition remains comparable to that of its wrought counterpart. Similarly, for AM IN718 alloys, HIP yields only a marginal improvement in fatigue life, with the resulting performance also approaching that of wrought material. This consistent trend stems from the negligible impact of process-induced porosity on fatigue in both AM IN625 and IN718. Their fatigue failures are instead dominated by other microstructural features [59]. For example, the rejuvenation heat treatment protocol for directionally solidified (DS) nickel-based superalloys in power generation gas turbines consists of three critical stages: (1) full solution treatment at 1225 °C for 4 h, (2) primary aging at 1120 °C for 2 h, and (3) secondary aging at 850 °C for 24 h.

Therefore, measures are usually taken to relieve residual stress or further improve the mechanical performance of remanufactured blades. Our laboratory developed several heat treatment methods, which were validated through field testing. The results further confirm the effectiveness of this remanufacturing approach for gas turbine blades.

## 6. Comparison with Other Remanufacturing Techniques

**Figure 15:**
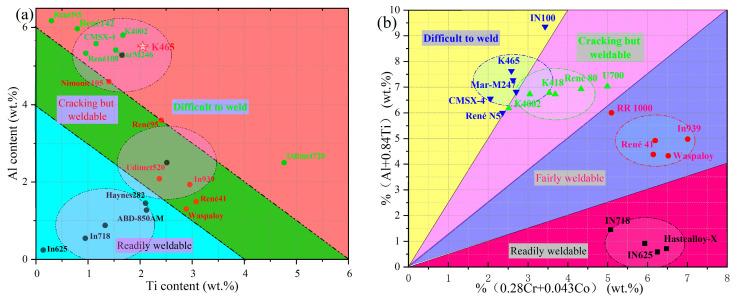
Relationship between the weldability assessment diagrams and alloying elements in Ni-based superalloys: (**a**) SAC based on the Ti vs. Al diagram and (**b**) DDC considering the effects of Cr and Co (adapted from ref. [60]).

As-stated in the previous section once reported, the chemical composition of K 465 is (9.5–11%) W, 8–9.5% Cr, (5.1–6%) Al, (1.5–2.5%) Ti, (9–10.5%) Co, (1.2–2.4%) Mo, (0.8–1.2%) Nb, ≤0.035% B, and ≤0.04% Zr; it is balanced using Ni. A chemical composition of 0.14 C, 8.30 Cr, 9.45 CO, 1.78 Mo, 9.85 W, 5.56 Al, 2.38 Ti, 0.90 Nb, B ≤ 0.035, and Zr ≤ 0.04, with Ni balance, is selected for the purpose of discussion. K46. The location is shown in Figure 15. Total Al + Ti content exceeds 6 wt%, and Al + 0.84%Ti (in wt%) versus 0.28%Cr + 0.043%Co. It is highly susceptible to ductility-dip cracking (DDC) and is one of the most difficult-to-weld Ni-based superalloys. As illustrated in Figure 15 [60], alloys exhibiting a combined aluminum (Al) and titanium (Ti) content exceeding 4.5 wt% are typically classified as “unweldable”.

Repairing Ni-based superalloys presents significant challenges, primarily their susceptibility to HAZ and weld metal cracking [60]. Such cracking can initiate during fabrication, post-weld heat treatment, or in service. A primary factor contributing to this cracking tendency is the high γ’ fraction, which is achieved through the addition of Ti and Al. This elevated γ’ content increases the propensity for “strain-age cracking” (SAC), particularly during PWHT or in multi-pass welding scenarios [60]. Consequently, the total content of Al and Ti serves as an empirical criterion for assessing weldability. As illustrated in Figure 15a, superalloys with a combined Al + Ti content exceeding 6 wt% are generally deemed non-weldable [60].

The quality and performance of welded structures in advanced manufacturing are predominantly governed by the employed welding technique. Numerous studies affirm that the process parameters inherent to welding directly dictate critical outcomes, including joint quality, production throughput, and the resultant mechanical properties of the assembly [17]. Plasma Transfer Arc Welding (PTAW) is a widely adopted welding process renowned for its high production efficiency. Furthermore, it is characterized by enhanced shielding efficacy, exceptional arc stability, and a high deposition rate, which collectively contribute to its superior weld quality and operational productivity. Laser metal deposition (LMD) is an established additive manufacturing methodology, predominantly utilized for the repair and fabrication of intricate metallic components. The process operates by precisely delivering feedstock material into a concentrated heat source (e.g., arc, laser, or electron beam), generating a localized melt pool and facilitating the layer-wise deposition of material onto a substrate. Owing to its operational flexibility, LMD has garnered considerable interest for integration into diverse industrial workflows, with prominent applications encompassing component repair, surface cladding, and near-net-shape manufacturing. The selection between Plasma Transfer Arc Welding (PTAW) and Laser Deposition for blade repair involves a trade-off between cost, performance, and application specificity. For non-critical structural repairs or cladding large volumes where productivity is paramount, PTAW offers a robust and economical solution. For critical, fatigue-sensitive areas requiring precise geometry control and minimal thermal impact, Laser Metal Deposition is the technologically superior choice, despite its higher initial cost. Then, for the non-weldable K 465 alloy repair, laser metal deposition is the optimal choice.

Electron beam additive manufacturing deposits layers using wire feedstock or powder and an electron gun as the energy source. A comparative analysis of the non-weldable superalloy IN738 reveals a stark contrast in recrystallization resistance between components fabricated via laser-directed energy deposition (DED) without preheating and those produced by electron beam powder bed fusion (EB-PBF). The EB-PBF process, which employs in situ preheating to the upper ductility dip temperature range, yields an as-printed microstructure with minimal stored energy, thereby conferring exceptional stability against recrystallization [61]. The non-weldable nickel-based superalloy was fabricated by selective electron beam melting (S-EBM). Its characterization revealed a heterogeneous microstructure containing processing-induced defects [62]. These included gas porosity originating from the powder feedstock, as well as shrinkage pores and solidification cracks formed during S-EBM. Investigation into crack pathogenesis highlighted a consistent intergranular fracture mode. Statistical analysis demonstrated a strong association between crack propagation paths and high-angle grain boundaries (HAGBs), with crystallographic misorientation being a key influencing factor. Fractography of as-built micro-tensile specimens confirmed the cracks as hot tears [62,63].

But, unfortunately, Electron Beam-Directed Energy Deposition (EB-DED), while advantageous for its high-power density and vacuum environment, faces significant challenges in welding and repairing superalloys, particularly those prone to strain-age cracking. The primary limitations include:

**※ High Heat Input and Thermal Stress**: The concentrated energy source generates steep thermal gradients and rapid cooling rates, inducing significant residual stresses. This can exceed the strain tolerance of precipitation-strengthened superalloys, especially in the heat-affected zone (HAZ).

**※ Susceptibility to Cracking**: High residual stress combined with strengthening phase (e.g., γ′) precipitation during thermal cycles can cause solidification cracking in the fusion zone. More critically, this combination also induces strain-age cracking in the HAZ of non-weldable or low-ductility superalloys.

**※ Microstructural Inhomogeneity**: Achieving a consistent, defect-free microstructure that matches the base material’s properties is challenging. Issues such as element segregation, formation of brittle phases, and undesirable grain growth can compromise the mechanical integrity of the repaired component.

**※ Process Complexity for Repair**: Successful repair requires precise control over pre-heating, deposition parameters, and post-process heat treatment to mitigate stresses and restore microstructure. This necessity makes the process highly complex and parameter-sensitive for high-integrity applications.

## 7. Conclusions and Future Trends

Laser metal additive remanufacturing technologies have emerged as a revolutionary approach for the development of innovative methods of turbine blade repair. The main conclusions and future trends can be summarized as follows:

**(a) Main Contributions: Current Advances in Laser Metal Deposition for Superalloy Repair**

Laser metal additive remanufacturing is now an established transformative technology for repairing damaged superalloy turbine blades. It has achieved substantial progress in both technical development and industrial application. Its core contributions are multifaceted.

First, industrial validation of long-term applicability has been achieved. Laser additive remanufacturing has proven to be a mature and reliable method for repairing superalloy gas turbines. This is supported by nearly 30 years of industrial application at Wuxi Zhongke Jinyan, where it has successfully remedied corrosion, wear, and surface spalling in high-temperature core components.

Second, material–process synergy has been realized through targeted innovation. Researchers have developed specialized alloy powders tailored to the characteristics of laser additive processes, ensuring compatibility between the deposited material and the superalloy substrate. This addresses the long-standing challenge of material mismatching, which previously led to interfacial cracking or performance degradation. A novel laser additive remanufacturing process has been developed based on the unique metallurgical properties of high-temperature alloy turbine blades. This process optimizes parameters like laser power and scanning speed to control molten pool behavior.

Third, preliminary intelligent frameworks have been developed for laser additive manufacturing of high-temperature alloy blades. By integrating deep learning, they enable multi-objective cooperative process optimization. This moves beyond empirical parameter tuning, laying the groundwork for balancing repair accuracy, material density, and mechanical performance—key metrics for ensuring repaired components meet service requirements.

Collectively, these advances establish laser metal deposition as a superior repair method. It restores superalloy components to near-original performance while reducing replacement costs by over 30%.

**(b) Clear Limitations of Current Research and Applications**

Despite significant progress, the application of laser metal deposition in superalloy turbine blade repair remains constrained by technical and practical bottlenecks:

**(** **1) Immature process control leading to performance inconsistencies**

LMD technology for turbine blade repair is still in its early stages, with limited understanding of the “process–microstructure–performance” relationship. Variations in the temperature gradient (G) and solidification rate (V) during molten pool evolution can produce uneven grain structures. For instance, epitaxial columnar grains introduce strong anisotropy, which can reduce lateral creep life by up to 60%. Improper energy density frequently causes defects like lack-of-fusion holes or keyhole porosity. These defects significantly compromise the reliability of repaired blades.

**(2) Inadequate post-repair processing and inspection systems**

Current research lacks tailored post-repair workflows. High-temperature alloys repaired via LMD often require subsequent heat treatment. This process eliminates high residual stresses—which can reach 420 MPa in as-printed parts—and regulates second-phase precipitation. For instance, it dissolves harmful Laves phases that can cause a 40% loss in tensile ductility. However, standardized heat treatment protocols for specific repair scenarios are not yet established. Additionally, rapid non-destructive testing (NDT) technologies are lacking. Conventional methods often fail to detect submicron cracks or internal porosity in complex blade geometries.

**(3) Limited adaptability to diverse repair scenarios**

The lack of versatile fixtures hampers precise alignment during LMD repair. This limitation is particularly evident when handling blades with non-standard damage or from different equipment models. Post-wire-cutting re-scanning protocols remain underdeveloped. This leads to incomplete damage characterization and imprecise material deposition. Consequently, repaired components may suffer from mismatched aerodynamic profiles or insufficient load-bearing capacity.

**(4) Scarcity of long-term service data**

Industrial applications have not accumulated sufficient long-term performance data for repaired components. Critical metrics remain unvalidated, including creep resistance at 650–800 °C and high-cycle fatigue life. This creates uncertainty for aerospace and energy applications requiring decades of reliable service.

**(c) Concrete Perspectives for Future Developments**

Addressing these limitations demands interdisciplinary collaboration. Key areas include materials science, artificial intelligence, and industrial engineering. Key future directions include:

**(1) Intelligent process optimization driven by machine learning**

AI can correlate energy density with defect formation, automatically adjusting parameters to prevent porosity. An energy density of 80–100 J/mm^3^ is optimal for achieving >99.5% density. Integrating in situ monitoring, such as thermal imaging of molten pools, with AI will enhance process stability. This approach can reduce performance variability by 40–50%.

**(2) Development of hybrid inspection and post-repair technologies**

Implementing “pre-repair characterization + in-process monitoring + post-repair verification” hybrid inspection systems is critical. Pre-repair, high-resolution 3D scanning (coupled with tailored re-scanning protocols) will enable precise mapping of damage. In practice, ultrasonic and X-ray computed tomography (CT) can detect defects in real time. Post repair, combining laser polishing (to reduce surface roughness Ra from 25 µm to 5 µm) with HIP (Hot Isostatic Pressing) treatment will close micro-pores and improve fatigue life by 5×.

**(3) Establishment of standardized industrial protocols**

Collaboration between academia and industry (e.g., MRO providers and OEMs) should yield unified standards covering alloy powder specifications, process parameters, heat treatment regimes, and quality inspection criteria. For instance, standardizing the “island scanning + substrate preheating (200 °C)” strategy can reduce residual stress by 30% across different repair facilities. Additionally, developing modular fixtures adaptable to diverse blade models will enhance alignment precision and operational efficiency.

**(4) Exploration of multi-material and functionalized repair**

Future research should focus on multi-material deposition to create gradient coatings (e.g., integrating wear-resistant nickel-chromium alloys with heat-resistant nickel-aluminum compounds to improve component durability). Simultaneously, embedding sensor arrays during repair will enable real-time monitoring of component health in service, facilitating predictive maintenance and extending operational lifespans.

## Data Availability

No new data were created or analyzed in this study. Data sharing is not applicable.

## Supplementary Materials

The following supporting information can be downloaded at: https://www.mdpi.com/article/10.3390/ma18245590/s1. Table S1: Ultimate Tensile Strength under different temperature for cast and heat treatment sample.
