# Supplementary material for: Gas Turbine Blade Failures Repaired Using Laser Metal Additive Remanufacturing
Source: Materials (Basel). 2025 Dec 12;18(24):5590. doi: 10.3390/ma18245590 (PMC12734870; doi:10.3390/ma18245590)
Supplement: Supplementary file 1 [file materials-18-05590-s001.zip › materials-3762487-supplementary.pdf]

materials MDPI

Supplementary Information

A Glossary

1. **Repair**

   The process of restoring a damaged component to an operational condition by addressing specific defects or failures, while maintaining the original design intent and material composition. In aircraft engine maintenance, this typically involves localized interventions such as crack repair, coating restoration, or dimensional recovery through techniques like laser cladding or welding.

2. **Remanufacturing**

   A comprehensive industrial process that returns used components to like-new condition through disassembly, cleaning, inspection, and replacement of worn parts, combined with advanced surface engineering technologies. For turbine blades, this involves complete stripping of coatings, metallurgical restoration, and application of new thermal barrier coatings (TBCs) to meet original equipment manufacturer (OEM) specifications.

3. **Rejuvenation**

   A specialized maintenance procedure that extends service life by reversing age-related degradation mechanisms without full disassembly. In compressor and turbine applications, this includes microstructural restoration through heat treatments (e.g., hot isostatic pressing), oxide layer removal, and surface reconditioning to recover mechanical properties diminished by thermal cycling and creep.

Key distinguishing characteristics :

- **Repair**: Targets specific damage (localized intervention)
- **Remanufacturing**: Achieves complete performance reset (systematic reconstruction)
- **Rejuvenation**: Focuses on material property recovery (mainly through microstructural regeneration)

B Mechanical properties of base materials K 465 alloy:

Table S1. Ultimate Tensile Strength under different temperature for cast and heat treatment sample.

Sample Satate	Temperature/℃	Ultimate Tensile Strength /MPa
Precision cast Test Block	20	1009~1017
	800	1020~1010
	900	790~815
	1000	555~585
Standard heat treatment Test Block	20	974.5
	600	1051.2
	700	1031.6
	800	1026.6
	900	826
	1000	557

C Experimental Procedures for Creep Test and Creep Behavior Characterization

C1Thermal Exposure and Creep Test Conditions

After the initial specimen preparation (detailed in Figure.13), the as-fabricated samples were subjected to isothermal thermal exposure treatments at two distinct temperatures: 1123 K (850 °C) and 1173 K (900 °C), with the maximum exposure duration extended up to 5000 h to simulate long-term high-temperature service environments.

Uniaxial tensile creep tests were conducted under constant load conditions in a static air atmosphere, in compliance with the international standard ISO 204:2009 (Metallic materials—Uniaxial creep testing method in tension). For the long-term creep performance evaluation, a specific test condition of 1123 K (850 °C)/280 MPa was selected, which is representative of the service stress-temperature regime for the target superalloy. All creep tests were performed using an universal electronic high-temperature creep and rupture testing machine (manufacturer: CRIMS（Changchun Research Institute for Mechanical Science Co., Ltd.），Model:RDL5050KN, Changchun, China), which enables real-time monitoring and control of load and temperature.

To ensure uniform temperature distribution within the critical gauge section of the specimens (The samples are 78 mm in total length with a gauge length of 25 mm, and a diameter of 5 mm.), a multi-point temperature monitoring system was implemented: in addition to the primary thermocouple (type K, calibration accuracy: ±0.5 °C) used for feedback control of the central heating zone of the gauge section, two auxiliary K-type thermocouples were affixed to the upper and lower regions of the gauge length. The temperature difference across the entire gauge section was maintained within ±2 °C throughout the tests, in accordance with the precision requirements specified in ISO 204:2009. For each creep test condition, duplicate experiments (n=2) were carried out to ensure the reproducibility of the test results, and the average values of the measured parameters were reported unless otherwise stated.

C2 Creep Lifetime Results and Analysis

The creep lifetime statistics of the superalloy samples under different thermal exposure conditions, and the key data are summarized in the following. The as-deposited (as-fabricated) samples exhibited an average creep lifetime of 5286.5 ± 246.5 h under the test condition of 1123 K (850 °C)/280 MPa, indicating excellent initial long-term creep resistance.

However, the creep lifetime of the samples decreased significantly after isothermal thermal exposure, and this degradation effect was strongly dependent on the exposure temperature. For the samples thermally exposed at 850 °C (1123 K), their creep lifetime was reduced by approximately 33% (i.e., one-third) compared to the as-deposited counterparts, suggesting that even moderate long-term thermal exposure can induce microstructural evolution (e.g., precipitate coarsening, grain boundary segregation) that impairs creep performance.

A more drastic reduction in creep lifetime was observed for the samples exposed to 900 °C (1173 K): after thermal exposure at this temperature, the average creep lifetime dropped to 1378 ± 233 h. Notably, with further extension of the 900 °C thermal exposure duration (up to 5000 h), the creep lifetime underwent a catastrophic decline, ultimately falling to a mere 78.5 ± 13.5 h. This significant lifetime degradation at 900 °C is attributed to the accelerated microstructural degradation mechanisms (e.g., excessive growth of γ' precipitates, formation of detrimental intermetallic phases, or grain boundary oxidation) under higher thermal exposure temperatures, which severely weaken the load-bearing capacity of the alloy during creep deformation.
